# Ultra-Processed Food Intake Is Associated with Dietary Quality, Dental Caries, and Constipation-Related Symptoms Among Preschool Children in Taiwan: A Cross-Sectional Study

**DOI:** 10.3390/nu18213670

**Published:** 2026-11-05

**Authors:** Shu-Chi Mu, Sing-Chung Li, Shan-Ching Hsu, Yi-Ling Chen, Yu-Tung Chang, Shu-Fen Liao, Chiao-Ming Chen

**Affiliations:** 1 Department of Pediatrics, Shin-Kong Wu Ho-Su Memorial Hospital, Taipei 11101, Taiwan; musc1004@gmail.com (S.-C.M.); ylchen1219@gmail.com (Y.-L.C.); 2 School of Medicine, College of Medicine, Fu-Jen Catholic University, New Taipei City 24205, Taiwan; 3 School of Nutrition and Health Sciences, College of Nutrition, Taipei Medical University, Taipei 11031, Taiwan; sinchung@tmu.edu.tw; 4 Department of Food Science, Nutrition, and Nutraceutical Biotechnology, Shih Chien University, Taipei 10462, Taiwan; helen1016@g2.usc.edu.tw (S.-C.H.); sa030520@gmail.com (Y.-T.C.); 5 Department of Education, Shin Kong Wu Ho-Su Memorial Hospital, Taipei 11101, Taiwan; a010866@ms.skh.org.tw

**Keywords:** ultra-processed foods, preschool children, dental caries, constipation, dietary quality

## Abstract

**Background/Objectives:** Evidence on ultra-processed food (UPF) consumption and health among East Asian preschoolers is limited. We examined associations with nutrient intake, anthropometry, dental caries, and bowel symptoms in Taiwan. **Methods:** This cross-sectional study included 822 children aged 2–6 years. A caregiver-reported 107-item food frequency questionnaire estimated UPF energy contribution using an adapted NOVA framework; anthropometric and dental data were available for 777 and 746 children, respectively. **Results:** UPFs supplied 15.12% of energy. Higher UPF intake was associated with lower fiber, protein, and several micronutrient intakes and higher saturated fat and sodium intakes, without significant anthropometric differences. Caries prevalence was 12.8% in Q1 and 31.2% in Q4 (adjusted prevalence ratio [aPR] = 2.27, 95% confidence interval [CI]: 1.48–3.48). Relative to Q1, Q3 showed higher prevalences of infrequent bowel movements (aPR = 1.48, 95% CI: 1.09–2.00) and hard stools (aPR = 1.23, 95% CI: 1.01–1.49); Q4 associations were not statistically significant. Exploratory food-group analyses associated fruit intake with lower reported frequency of infrequent bowel movements and biscuit/snack intake with higher hard stool frequency. **Conclusions:** Higher UPF intake was associated with unfavorable nutrient profiles, dental caries, and selected caregiver-reported bowel symptoms, but not anthropometric status. Further longitudinal studies are warranted to clarify the temporal relationships between UPF consumption and these health outcomes during early childhood.

## 1. Introduction

Ultra-processed foods (UPFs) have become an important dietary exposure in modern food environments. According to the NOVA food classification system, foods are categorized based on the extent and purpose of industrial processing rather than solely on nutrient composition. UPFs are industrial formulations made mostly from substances extracted or derived from foods, together with additives used to improve flavor, color, texture, shelf life, and palatability. These products usually contain little or no intact whole food and commonly include sugar-sweetened beverages, packaged snacks, confectionery, instant noodles, processed meat products, and ready-to-eat or ready-to-heat meals [1]. A practical way to identify UPFs is the presence of ingredients rarely used in home cooking, such as high-fructose corn syrup, hydrogenated oils, hydrolyzed proteins, modified starches, flavor enhancers, emulsifiers, sweeteners, thickeners, and coloring agents [1].

The consumption of UPFs has increased globally and is regarded as a key feature of the nutrition transition. In general, countries with more industrialized food systems and higher availability of packaged and convenience foods tend to have greater dietary contributions from UPFs. A systematic review of worldwide UPF consumption reported particularly high contributions of UPFs to total energy intake in high-income Western countries, including the United States and the United Kingdom [2]. Across European countries, the proportion of energy intake from ultra-processed foods and drinks varies substantially, ranging from approximately 14% to 44%, with higher levels reported in countries such as the United Kingdom and Sweden [3]. In East Asia, evidence also indicates that UPF consumption is no longer limited to Western dietary patterns. In Japan, UPFs contributed 38.2% of total daily energy intake among middle-aged adults, and highly processed food consumption among Japanese children and adolescents has been associated with poorer overall diet quality [4,5]. In Korea, national data showed that UPF intake among children and adolescents increased over time, with preschool-aged children showing an increase from 23.5% to 26.4% of total energy intake between 2007 and 2024 [6]. In China, studies based on national survey data suggest that UPF consumption among children and adolescents is emerging as a relevant dietary concern and has been associated with overweight and obesity [7].

UPF exposure begins early in life and may influence dietary patterns during childhood. In a community-based study of children aged 3–5 years in the United States, UPFs accounted for 67.6% of total caloric intake, indicating that UPF consumption can be very high even during the preschool period [8]. In the United Kingdom Gemini cohort, the contribution of UPFs to total energy intake was 46.9% at 21 months of age and increased to 59.4% by 7 years of age, suggesting that high UPF intake may track from toddlerhood into later childhood [9]. In Canadian preschoolers, higher UPF intake was associated with family and environmental factors, including parental UPF consumption, greater screen time, and reduced access to fresh food markets [10]. Studies from Brazil have also reported early and frequent exposure to UPFs among young children, with national data showing high prevalence of UPF consumption among children under 5 years of age [11,12]. These findings suggest that early childhood is a critical period for understanding UPF exposure and its potential health implications.

Accumulating evidence has linked higher UPF consumption with multiple adverse health outcomes. Umbrella reviews of epidemiological meta-analyses have reported positive associations between UPF exposure and all-cause mortality, cardiovascular disease, type 2 diabetes, obesity, metabolic syndrome, depression, sleep-related outcomes, gastrointestinal outcomes, and some cancers [13,14]. A randomized inpatient feeding trial further demonstrated that an ultra-processed diet caused higher ad libitum energy intake and weight gain compared with an unprocessed diet, even when the diets were matched for presented calories, macronutrients, sugar, sodium, and fiber [15]. Possible mechanisms include high energy density, high palatability, rapid eating rate, excessive intake of added sugars, saturated fat, and sodium, low dietary fiber and micronutrient density, displacement of minimally processed foods, disruption of the food matrix, and exposure to additives or packaging-related contaminants [13,14,15].

For preschool children, UPF intake is particularly concerning because this developmental stage is important for establishing food preferences, dietary habits, oral health, bowel habits, and long-term metabolic risk. UPFs may reduce overall dietary quality by displacing fruits, vegetables, whole grains, legumes, dairy products, and other nutrient-dense foods. They may also increase exposure to free sugars, refined starches, sodium, and saturated fat. In relation to oral health, a systematic review and meta-analysis reported that UPF consumption was associated with dental caries in children and adolescents, with stronger evidence among studies involving children younger than 6 years [16].

A study of children aged 0–3 years in Southern Brazil also examined UPF consumption in relation to early childhood caries, supporting the relevance of UPF exposure to oral health during early life [17]. In addition, because UPF-rich diets tend to be lower in dietary fiber and micronutrients, they may plausibly influence stool consistency and constipation-related symptoms, although evidence focusing specifically on preschool-aged children remains limited.

In Taiwan, research directly examining UPF intake and related health outcomes remains limited. A study using the Nutrition and Health Surveys in Taiwan reported that among Taiwanese adolescents aged 16–18 years, the contribution of UPFs to total energy intake increased from 21% in 1993–1996 to 25% in 2011, accompanied by a decline in energy from original foods and poorer dietary quality [18]. Another Taiwanese study applied the NOVA classification to NAHSIT data and examined food processing levels in relation to urinary phthalate metabolites, suggesting that the degree of food processing may also be relevant to environmental chemical exposure [19]. More recently, a study using NAHSIT 2017–2020 data from 6538 Taiwanese adults suggested that replacing UPFs with less-processed food alternatives could improve dietary intakes of calcium, zinc, and dietary fiber [20]. However, these studies primarily focused on adolescents or adults, and contemporary evidence regarding UPF consumption among Taiwanese preschool children remains scarce. In particular, the contribution of UPFs to total energy intake and their associations with dietary quality and health indicators in this age group have not been well characterized.

Therefore, an important research gap remains. The UPF intake profile of Taiwanese preschool children is still unclear, and its potential associations with nutrient intake, anthropometric status, dental caries, and bowel habits have not been adequately investigated. Given that preschool age is a critical period for the development of dietary behaviors and health-related habits, understanding UPF intake in this population is essential for early dietary guidance and public health intervention. In Taiwan, routine preschool health screening programs provide a multidimensional assessment of children’s health. For example, the Taipei City Integrated Community Screening Program for Preschool Children includes assessments of growth and weight status, oral health, vision, and hearing. This public health framework, together with previous evidence suggesting associations between UPF consumption and dietary quality, adiposity-related outcomes, and oral health, motivated us to investigate whether UPF intake is associated with selected health indicators among preschool children. Accordingly, the present study aimed to examine the associations of UPF intake with demographic characteristics, family dietary habits, nutrient intake, anthropometric status, dental caries, and constipation-related symptoms. Although these indicators represent distinct health domains, they provide complementary information on early-childhood nutritional health: nutrient intake reflects dietary quality; anthropometric status indicates growth and weight status; dental caries reflects oral health; and constipation-related symptoms provide information on gastrointestinal function. Examining these indicators together may provide a broader understanding of the health correlates of UPF consumption during early childhood.

## 2. Materials and Methods

### 2.1. Study Design

This study adopted a cross-sectional design combining questionnaire survey data with preschool children’s health examination data. The study aimed to investigate the association between ultra-processed food (UPF) intake and selected health indicators among preschool children in Taiwan.

This study was conducted in collaboration with the “Taipei City Integrated Community Screening Service Program for Preschool Children.” The study protocol was reviewed and approved by the Institutional Review Board (IRB) of Shin Kong Wu Ho-Su Memorial Hospital (approval number 20230415R; approval date: 13 July 2023). All study procedures complied with ethical regulations. Participation was completely voluntary, and parents or primary caregivers provided online informed consent before completing the questionnaire.

### 2.2. Study Participants and Recruitment

Participant recruitment was conducted from March to August 2024 in conjunction with the health screening program carried out by the pediatric medical team of Shin Kong Wu Ho-Su Memorial Hospital. The target population consisted of preschool children aged 2–6 years attending 82 registered kindergartens in Shilin District, Taipei City, including both public and private kindergartens. All kindergartens participating in the preschool health examination program were invited to take part in the study, without random selection. Participation at the individual child level was voluntary, and parents or legal guardians independently decided whether their children would participate.

During the health screening period, trained research assistants or healthcare professionals explained the study purpose, participation procedures, questionnaire contents, confidentiality measures, and participants’ rights to homeroom teachers and kindergarten staff through both oral and written instructions. The teachers then assisted in distributing the online questionnaire link and written study information sheet to parents or primary caregivers. Parents were informed that participation was entirely voluntary and that they could withdraw from the study at any time without any adverse consequences. Parents could complete the questionnaire using a mobile phone or other electronic devices. The average completion time was approximately 15–20 min. Submitted questionnaire responses were directly stored in a secure and encrypted research database for subsequent data management and analysis.

### 2.3. Basic Information Collection

Basic information, including children’s age, sex, and household food-related practices, was collected using an online structured questionnaire completed by parents or primary caregivers.

Household food-related practices were assessed using three questionnaire items referring to the preceding month: frequency of home cooking, frequency of purchasing frozen foods from supermarkets or convenience stores for the child, and frequency of purchasing refrigerated ready-to-eat foods from convenience stores for the child. These items were intended to characterize household food preparation and purchasing practices and were assessed separately from the NOVA-based food classification. The purchasing frequencies of frozen and refrigerated ready-to-eat foods were not used to determine the NOVA processing categories.

Questionnaire data were de-identified for statistical analysis. Personal identifying information was stored separately from research data to protect participant confidentiality.

### 2.4. Dietary Assessment and Classification of Ultra-Processed Foods

Habitual dietary intake was assessed using a semi-quantitative food frequency questionnaire (SQ-FFQ) developed for preschool children. The questionnaire was completed by the primary caregiver based on the child’s dietary intake during the preceding month. The SQ-FFQ was designed collaboratively by nutritionists and epidemiological researchers and was adapted to reflect commonly consumed foods and dietary patterns in Taiwan. Information on food items, intake frequency, and portion size was collected.

The SQ-FFQ included 107 commonly consumed food items in Taiwan. Based on the NOVA food classification framework and Taiwanese dietary characteristics, all food items were classified into four groups according to the degree and purpose of food processing. In the original NOVA classification, Group 2 consists of processed culinary ingredients, such as oils, salt, and sugar, which are generally consumed as components of prepared foods rather than as individual food items. Because these ingredients are difficult to assess separately using a food-based SQ-FFQ, we adopted a modified NOVA-based classification framework to better reflect the structure of the questionnaire and commonly consumed foods in Taiwan. The first group comprised unprocessed or minimally processed foods, including rice, vegetables, fruits, fresh milk, eggs, fish, meat, tofu, and nuts (32 items). The second group included processed culinary ingredients and selected moderately processed foods, such as convenience store lunch boxes, sushi, and packaged bread (15 items). The third group comprised processed foods, including canned vegetables, canned fruits, pickled vegetables, sweetened dried fruits, and surimi-based products (19 items). The fourth group included ultra-processed foods and beverages, such as instant noodles, sugar-sweetened beverages, snacks, candies, and cookies (41 items). Although the classification of Groups 2 and 3 differs from the original NOVA framework, the primary exposure variable in this study was defined exclusively by the proportion of total energy intake derived from Group 4 (ultra-processed foods). Therefore, differences in the classification of foods between Groups 2 and 3 did not affect the calculation of UPF energy contribution, provided that the classification of Group 4 foods remained unchanged.

All 107 food items were independently evaluated and classified by two nutrition researchers using predefined criteria based primarily on the degree and purpose of industrial processing, including the presence of industrial formulations, additives, and ingredients not commonly used in home cooking, rather than solely on nutrient composition. Following the independent assessments, any discrepancies in classification were resolved through discussion until consensus was reached. Subsequently, the consensus classifications were reviewed by two additional experts in pediatric nutrition and child health to assess their appropriateness and relevance to Taiwanese preschool children’s dietary patterns. The final food classifications were established following this review process. Formal inter-rater agreement statistics, such as Cohen’s kappa coefficient, were not calculated.

For each food item, intake frequency was recorded using eight response categories: never, once per month, 2–3 times per month, once per week, 2–3 times per week, 4–6 times per week, once per day, and 2–3 times per day. Portion sizes were estimated using semi-quantitative standards based on standard household containers, such as bowls, cups, and spoons, or countable units for specific food items.

Daily intake of each food item was estimated by multiplying the reported intake frequency by the corresponding portion size and converting it into average daily intake. Energy and nutrient intakes, including protein, fat, carbohydrate, vitamins, and minerals, were calculated by matching each food item with the corresponding nutrient composition values from the Taiwan Food Composition Database published by the Ministry of Health and Welfare, Taiwan, 2024 version.

The percentage of total energy intake derived from ultra-processed foods was calculated for each participant by dividing the total energy intake from Group 4 foods by total daily energy intake and multiplying by 100. Participants were then categorized into quartiles according to this percentage of total energy intake derived from ultra-processed foods.

### 2.5. Anthropometric Measurements

Anthropometric data were collected by the pediatric team of Shin Kong Wu Ho-Su Memorial Hospital during on-site health examinations following standardized procedures. Height and weight were measured using calibrated electronic height and weight measurement equipment to ensure the accuracy and consistency of the measurements. During the measurements, children were asked to remove their shoes, hats, and heavy outer clothing. They were instructed to stand upright in a natural posture, and the measurements were performed by trained healthcare professionals with medical backgrounds to ensure that the procedures complied with child growth assessment standards.

Height and weight percentiles were subsequently calculated according to sex- and age-specific Taiwanese child growth reference values [21]. Body mass index (BMI) was further calculated, and BMI status was classified into four categories—underweight, normal weight, overweight, and obesity—according to the standards announced by the Health Promotion Administration, Ministry of Health and Welfare, Taiwan [22].

### 2.6. Dental Caries Assessment

Dental caries status was assessed by dentists experienced in pediatric oral health examinations during the preschool health screening program. Caries status was determined through routine clinical oral examinations based on the dentists’ professional judgment.

The numbers of decayed primary teeth, decayed permanent teeth, filled primary teeth, and filled permanent teeth were recorded separately for each child. Because the study population included children aged 2–6 years, some older children may have entered the early mixed-dentition stage. Therefore, both primary and permanent teeth were included to assess overall dental caries experience.

Children were classified as having dental caries experience if at least one decayed or filled tooth was identified in either dentition. The total number of caries-affected teeth was calculated as the sum of decayed and filled teeth, including decayed primary teeth, decayed permanent teeth, filled primary teeth, and filled permanent teeth.

### 2.7. Bowel Habit Assessment

Bowel habits and constipation-related symptoms were assessed using six caregiver-reported items included in the study questionnaire. These items evaluated (1) infrequent bowel movements (fewer than three times per week), (2) straining during defecation, (3) hard or lumpy stools, (4) a sensation of incomplete evacuation, (5) a sensation of anorectal blockage, and (6) the need for manual assistance to facilitate defecation. Each symptom was rated using a five-point frequency scale: 0 = never or rarely, 1 = sometimes, 2 = often, 3 = most of the time, and 4 = almost always.

Caregivers were asked to report the frequency of these symptoms during the preceding three months. This recall period was selected to characterize recent bowel-habit patterns and reduce the influence of transient symptoms rather than to establish a clinical diagnosis.

To facilitate caregiver assessment of subjective bowel symptoms in young children, simplified operational definitions were provided. The sensation of incomplete evacuation was defined as the child’s expression of a persistent urge to defecate after completing a bowel movement, whereas the sensation of anorectal blockage was interpreted as the child’s expression of a desire to defecate accompanied by difficulty or an inability to pass stool. Caregivers assessed these symptoms based on their children’s verbal expressions and observed bowel habits. Because these assessments relied on caregiver perceptions rather than direct clinical evaluation, the resulting symptom scores were used to characterize constipation-related symptoms rather than to diagnose functional constipation.

### 2.8. Statistical Analysis

All statistical analyses were performed using IBM SPSS Statistics version 27.0 (IBM Corp., Armonk, NY, USA). Descriptive statistics were used to summarize participant characteristics, dietary intake, anthropometric measures, dental caries, and constipation-related symptoms. Continuous variables are presented as mean ± standard deviation (SD), whereas categorical variables are presented as numbers and percentages.

Participants were categorized into quartiles (Q1–Q4) according to the percentage of total energy intake derived from ultra-processed foods (UPFs). Differences in categorical variables, including sex, age group, anthropometric status, and dental caries status, across UPF intake quartiles were examined using the chi-square test. Differences in continuous variables, including energy and nutrient intakes, anthropometric measures, the number of dental caries-affected teeth, and constipation-related symptom scores, were examined using one-way analysis of variance (ANOVA). When the overall ANOVA was statistically significant, Scheffé’s post hoc test was performed for pairwise comparisons among quartiles.

Constipation-related symptoms were assessed using six caregiver-reported items, each rated on a five-point ordinal scale ranging from 0 to 4. The distributions of individual symptom scores across UPF intake quartiles were compared using Pearson’s chi-square test. Because some categories had low expected cell frequencies, Monte Carlo *p*-values were calculated to provide more reliable statistical inference. To account for multiple comparisons across the six symptoms, *p*-values were adjusted using the Benjamini–Hochberg false discovery rate (FDR) procedure.

For multivariable analyses, each constipation-related symptom was dichotomized as 0 (never or rarely) versus 1–4 (at least sometimes), according to the original questionnaire response categories. Modified Poisson regression with robust standard errors was used to estimate adjusted prevalence ratios (aPRs) and 95% confidence intervals (CIs) across UPF intake quartiles, with Q1 as the reference category. The models were adjusted for age group, sex, and frequency of home cooking. Linear trends across UPF intake quartiles were assessed by entering quartile categories as an ordinal variable in the corresponding regression models.

To further investigate the associations between specific food groups and bowel-related symptoms, the frequencies of infrequent bowel movements and hard stools were analyzed as separate ordinal outcomes using multivariable ordinal logistic regression. Daily intakes of whole grains, vegetables, fruit, legumes, dairy products, biscuits/snacks, and sugary foods/beverages were entered simultaneously into mutually adjusted models, with adjustment for age, sex, and total energy intake. Results were expressed as adjusted odds ratios (aORs) and 95% CIs, representing the odds of reporting a higher symptom-frequency category per unit increase in food group intake.

The primary constipation-related analyses focused on infrequent bowel movements and hard stool consistency, whereas the complete distributions and multivariable associations of all six symptoms were presented in the Supplementary Materials. These caregiver-reported measures were analyzed as individual symptoms rather than as a composite score or a clinical diagnosis of functional constipation.

To examine the independent associations of UPF intake with dental caries prevalence, multivariable regression analyses were performed using UPF intake quartiles as the exposure, with Q1 as the reference category. The primary models were adjusted for age group, sex, total energy intake, and frequency of home cooking. Dental caries prevalence was defined as the presence of at least one decayed or filled tooth and was analyzed using modified Poisson regression with robust standard errors to estimate adjusted prevalence ratios (aPRs) and 95% confidence intervals (CIs).

For the food group analyses of dental caries, dental caries prevalence was analyzed using modified Poisson regression with robust standard errors, with results expressed as adjusted prevalence ratios (aPRs) and 95% confidence intervals (CIs). The number of dental caries-affected teeth was analyzed as a count outcome using negative binomial regression, with adjusted incidence rate ratios (aIRRs) and 95% CIs reported. The total number of dental caries-affected teeth was calculated as the sum of decayed primary teeth, decayed permanent teeth, filled primary teeth, and filled permanent teeth. Effect estimates represented the relative change in dental caries prevalence or expected caries count associated with a one-serving/day increase in the corresponding food group while holding other food groups and covariates constant.

Multicollinearity among food group variables was assessed using the variance inflation factor (VIF). Because sugary snacks and sugar-sweetened beverages exhibited substantial multicollinearity when entered separately, they were combined into a single variable termed “sugary foods/beverages” in the final models. VIF values in the final models were all below 2 (range: 1.04–1.19), indicating no evidence of substantial multicollinearity. All statistical tests were two-sided, and a *p* value < 0.05 was considered statistically significant.

## 3. Results

### 3.1. Participant Selection and Quartile Grouping of Ultra-Processed Food Intake

**Figure 1:**
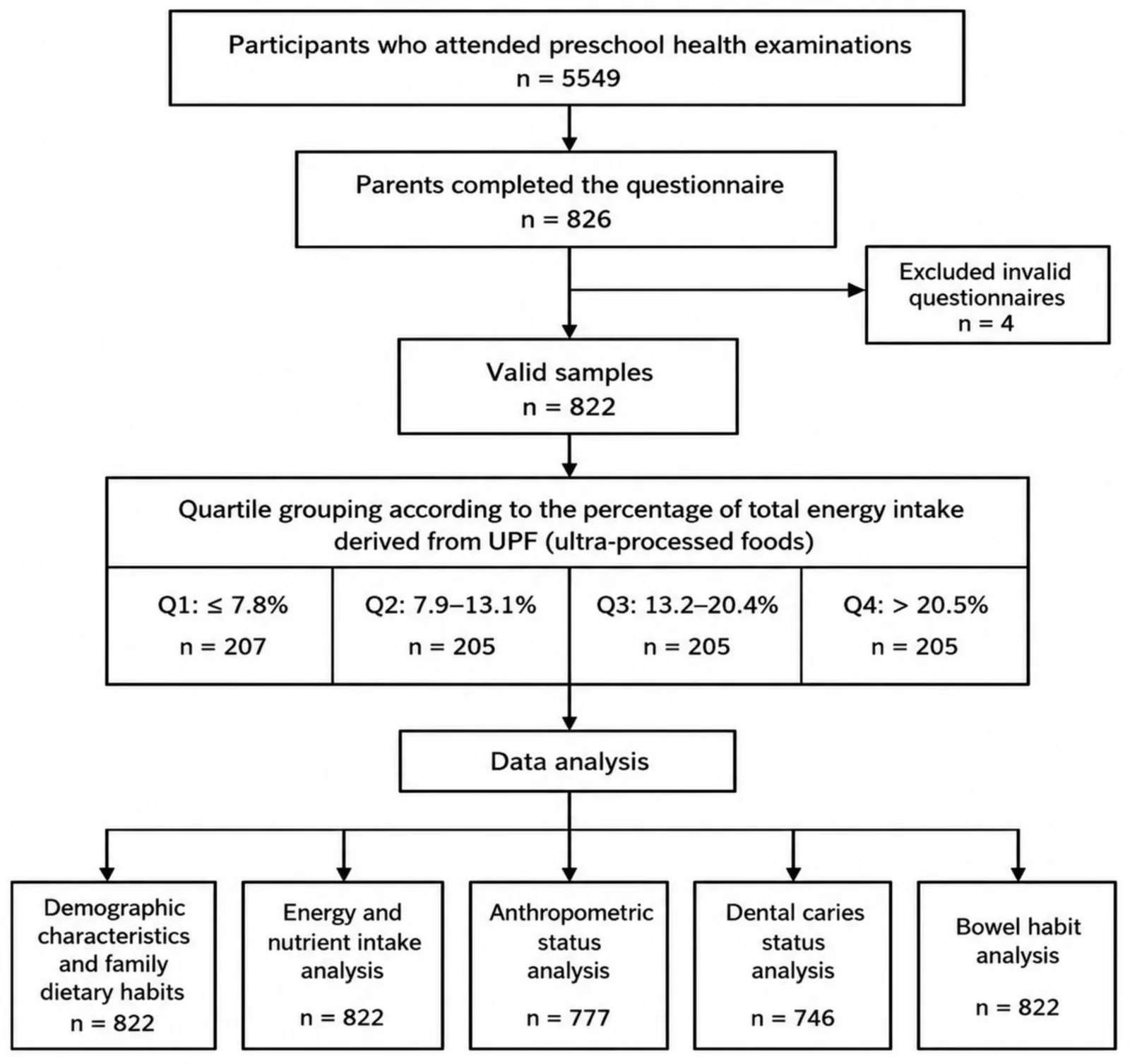
Flow Diagram of Participant Selection, UPF Intake Quartile Grouping, and Outcome-Specific Analyses.

A total of 5549 preschool children participated in the health examinations. Among them, 826 parents completed the questionnaire. After excluding 4 invalid questionnaires, 822 valid samples were included in the final analysis. The participant selection process and outcome-specific analytic samples are shown in Figure 1. The percentage of total energy intake derived from ultra-processed foods (UPFs) ranged from 0% to 88.9%. The 25th percentile, median, and 75th percentile values were 7.80%, 13.10%, and 20.43%, respectively, with a mean value of 15.12%. Based on the percentage of total energy intake derived from UPFs, participants were categorized into four quartiles: Q1, ≤7.8% (*n* = 207); Q2, >7.8% to ≤13.1% (*n* = 205); Q3, >13.1% to ≤20.4% (*n* = 205); and Q4, >20.4% (*n* = 205). Demographic characteristics, dietary intake, and bowel habit assessments were obtained from the parent-reported questionnaire; therefore, the analytic sample size for these analyses was 822. When questionnaire data were linked with children’s health examination records, some outcome-specific data were unavailable. Consequently, the final analytic sample sizes were 777 for anthropometric status and 746 for dental caries status.

### 3.2. Basic Characteristics and Family Dietary Habits

The basic characteristics of preschool children according to quartiles of UPF intake are shown in Table 1. There were no significant differences in sex distribution across UPF intake quartiles (*p* = 0.094). The proportion of boys tended to decrease from Q1 to Q4, whereas the proportion of girls tended to increase, but the difference was not statistically significant. Age distribution also did not differ significantly among the four groups (*p* = 0.154).

Regarding family dietary habits, the frequency of home cooking was similar across quartiles, with no significant difference among groups (*p* = 0.870). In contrast, the frequency of purchasing refrigerated ready-to-eat foods for the child increased significantly across UPF intake quartiles, from 0.5 ± 1.4 times/week in Q1 to 1.3 ± 2.5 times/week in Q4 (*p* < 0.001). Similarly, the frequency of purchasing frozen foods for the child increased from 0.4 ± 0.8 times/week in Q1 to 1.3 ± 2.9 times/week in Q4 (*p* < 0.001). Post hoc comparisons showed that Q4 had significantly higher consumption frequencies of both refrigerated ready-to-eat foods and frozen foods than Q1 and Q2.

### 3.3. Energy and Nutrient Intakes

Energy and nutrient intakes across quartiles of UPF intake are presented in Table 2. Total energy intake differed significantly among quartiles (*p* = 0.049); however, Scheffé post hoc comparisons showed no significant pairwise differences between groups. Carbohydrate intake per 1000 kcal also differed significantly across quartiles (*p* = 0.022), but no significant pairwise differences were observed in the post hoc analysis.

Several nutrient intakes showed clear differences according to UPF intake quartiles. Dietary fiber intake decreased progressively from 8.4 ± 3.2 g/1000 kcal in Q1 to 5.9 ± 2.3 g/1000 kcal in Q4 (*p* < 0.001). Protein intake also decreased from 41 ± 6 g/1000 kcal in Q1 to 35 ± 5 g/1000 kcal in Q4 (*p* < 0.001). In contrast, total fat intake increased from 28 ± 7 g/1000 kcal in Q1 to 32 ± 5 g/1000 kcal in Q4 (*p* < 0.001), and saturated fatty acid intake increased from 11 ± 4 g/1000 kcal in Q1 to 13 ± 3 g/1000 kcal in Q4 (*p* < 0.001).

For mineral intakes, sodium intake increased significantly with higher UPF intake, from 657 ± 199 mg/1000 kcal in Q1 to 849 ± 216 mg/1000 kcal in Q4 (*p* < 0.001). In contrast, potassium, calcium, magnesium, zinc, and phosphorus intakes were significantly lower in higher UPF intake quartiles (all *p* < 0.001). Iron intake did not differ significantly among groups (*p* = 0.093).

For vitamin intakes, retinol equivalents, vitamin B1, vitamin B2, vitamin B6, vitamin B12, and vitamin C differed significantly across UPF intake quartiles. In general, children in higher UPF intake groups had lower intakes of these vitamins. For example, vitamin C intake decreased from 78 ± 41 mg/1000 kcal in Q1 to 57 ± 29 mg/1000 kcal in Q4 (*p* < 0.001). No significant differences were observed for α-tocopherol equivalents (*p* = 0.113) or niacin (*p* = 0.196).

### 3.4. Anthropometric Status

Anthropometric outcomes are shown in Table 3. Among the 777 children with available anthropometric data, height percentile did not differ significantly across UPF intake quartiles (*p* = 0.290). Weight percentile was also similar among groups (*p* = 0.829). The distribution of anthropometric status, including underweight, normal weight, overweight, and obesity, was not significantly associated with UPF intake quartiles (*p* = 0.858). The prevalence of overweight and obesity did not show a clear increasing trend across higher UPF intake groups.

### 3.5. Dental Caries Status

Dental caries status across quartiles of UPF intake is shown in Table 4. Among the 746 children with dental examination data, the prevalence of dental caries experience increased across UPF intake quartiles. The proportion of children with dental caries experience was 12.8% in Q1, 20.7% in Q2, 25.5% in Q3, and 31.2% in Q4. The association between UPF intake quartiles and dental caries experience was statistically significant (*p* < 0.001).

The mean number of caries-affected teeth also differed significantly across UPF intake quartiles (*p* < 0.001). Scheffé’s post hoc comparisons indicated that children in Q4 had significantly more caries-affected teeth than those in Q1, whereas no other pairwise comparisons reached statistical significance. The mean number of caries-affected teeth increased from 0.3 ± 1.0 in Q1 to 1.1 ± 2.2 in Q4. These findings suggest that higher UPF intake was associated with a greater prevalence of dental caries experience and a higher number of caries-affected teeth among preschool children.

### 3.6. Bowel Habits and Constipation-Related Symptoms

The distributions of caregiver-reported infrequent bowel movements and hard or lumpy stools during the preceding 3 months across quartiles of ultra-processed food (UPF) intake are presented in Table 5. Each symptom was assessed using a five-point ordinal frequency scale ranging from 0 (never) to 4 (almost always).

The distribution of infrequent bowel movements (fewer than three times per week) did not differ significantly across UPF intake quartiles (Monte Carlo *p* = 0.430; FDR-adjusted *p* = 0.516). The proportion of children whose caregivers reported never or rarely experiencing infrequent bowel movements was 75.8% in Q1, 71.2% in Q2, 63.9% in Q3, and 67.3% in Q4.

In contrast, the distribution of hard or lumpy stool frequency differed significantly across UPF intake quartiles (Monte Carlo *p* = 0.004; FDR-adjusted *p* = 0.026). The proportion of children whose caregivers reported hard or lumpy stools as occurring often was higher in Q4 (13.2%) than in Q1 (4.3%). Conversely, the proportion reporting never experiencing hard or lumpy stools was 54.1% in Q1 and 46.3% in Q4.

These findings indicate that UPF intake was associated with differences in the frequency distribution of caregiver-reported hard or lumpy stools, whereas no significant differences were observed for infrequent bowel movements.

### 3.7. Multivariable Associations of UPF Intake with Dental Caries and Constipation-Related Symptoms

In modified Poisson regression analysis with robust standard errors, higher UPF intake remained significantly associated with dental caries prevalence after adjustment for age group, sex, total energy intake, and frequency of home cooking (Table 6). Compared with children in the lowest UPF intake quartile (Q1), those in Q3 and Q4 had significantly higher prevalence of dental caries, with adjusted prevalence ratios (aPRs) of 1.960 (95% CI: 1.250–3.050; *p* = 0.003) and 2.270 (95% CI: 1.480–3.480; *p* < 0.001), respectively. The association for Q2 did not reach statistical significance (aPR = 1.580, 95% CI: 0.997–2.500; *p* = 0.052). A significant increasing trend in dental caries prevalence was observed across increasing UPF intake quartiles (*p* for trend <0.001).

The multivariable associations between ultra-processed food (UPF) intake quartiles and caregiver-reported infrequent bowel movements and hard stool symptoms are presented in Table 7. Modified Poisson regression analyses were performed with adjustment for age group, sex, and frequency of home cooking.

For infrequent bowel movements (fewer than three times per week), the prevalence increased from 24.2% in Q1 to 36.1% in Q3 and 32.7% in Q4. Compared with children in Q1, those in Q3 had a significantly higher prevalence of infrequent bowel movements (aPR = 1.48, 95% CI: 1.09–2.00; *p* = 0.012). Although the adjusted prevalence was also higher in Q4, the association did not reach statistical significance (aPR = 1.35, 95% CI: 0.99–1.83; *p* = 0.058). Nevertheless, a significant positive trend was observed across increasing UPF intake quartiles (*p* for trend = 0.019).

Similarly, the prevalence of hard or lumpy stools was higher in Q3 (55.1%) and Q4 (53.7%) than in Q1 (45.9%). After adjustment for potential confounding factors, children in Q3 had a significantly higher prevalence of hard or lumpy stools compared with those in Q1 (aPR = 1.23, 95% CI: 1.01–1.49; *p* = 0.037). The association for Q4 was positive but did not reach statistical significance (aPR = 1.21, 95% CI: 1.00–1.48; *p* = 0.056). However, a significant positive trend was observed across UPF intake quartiles (*p* for trend = 0.013).

Overall, these findings suggest that higher UPF intake was associated with a greater prevalence of certain caregiver-reported bowel-related symptoms, particularly infrequent bowel movements and hard or lumpy stools.

### 3.8. Associations of Food Group Intakes with Constipation-Related Symptoms and Dental Caries

Table 8 presents the associations between daily food group intakes and both caregiver-reported infrequent bowel movements and hard or lumpy stools using multivariable ordinal logistic regression. After adjustment for age, sex, total energy intake, and other food groups, higher fruit intake was significantly associated with lower odds of reporting more frequent episodes of infrequent bowel movements (aOR = 0.872, 95% CI: 0.801–0.948; *p* = 0.0014). No significant associations were observed for whole grains, vegetables, legumes, dairy products, biscuits/snacks, or sugary foods/beverages.

For hard or lumpy stools, higher biscuit/snack intake was significantly associated with greater odds of reporting higher symptom-frequency categories (aOR = 1.324, 95% CI: 1.073–1.634; *p* = 0.0090). Legume intake showed a nonsignificant inverse association (aOR = 0.479, 95% CI: 0.217–1.056; *p* = 0.0681), whereas no significant associations were observed for the other food groups.

These findings suggest that higher fruit intake was associated with less frequent reports of infrequent bowel movements, whereas higher biscuit/snack intake was associated with more frequent reports of hard or lumpy stools among preschool children.

Table 9 shows the multivariable associations between daily food group intakes and dental caries outcomes. Higher vegetable intake was significantly associated with a lower prevalence of dental caries. Each additional serving/day of vegetables was associated with a 19.0% lower prevalence of dental caries (aPR = 0.810, 95% CI: 0.660–0.990; *p* = 0.038) after adjustment for age, sex, total energy intake, and the other food groups included in the model. No statistically significant associations with dental caries prevalence were observed for whole grains, fruit, legumes, dairy products, biscuits/snacks, or sugary foods/beverages. When the number of dental caries-affected teeth was analyzed using negative binomial regression, none of the individual food groups showed a statistically significant independent association with caries count. Sugary foods/beverages showed a borderline positive association with caries count (aIRR = 1.540, 95% CI: 0.990–2.380; *p* = 0.054), but the association did not reach statistical significance.

## 4. Discussion

The present study investigated the associations between ultra-processed food (UPF) intake and dietary quality, dental caries, and constipation-related symptoms among preschool children in Taiwan. Three major findings were observed. First, higher UPF intake was associated with an unfavorable nutrient profile, characterized by lower intakes of dietary fiber, protein, and several micronutrients, together with higher intakes of total fat, saturated fatty acids, and sodium. Second, children with higher UPF intake had a substantially higher prevalence of dental caries after adjustment for age, sex, total energy intake, and home cooking frequency. Compared with children in the lowest UPF quartile (Q1), those in the highest quartile (Q4) had more than a two-fold higher prevalence of dental caries (aPR = 2.270, 95% CI: 1.480–3.480; *p* < 0.001). Third, significant positive trends were observed between increasing UPF intake quartiles and the prevalence of caregiver-reported infrequent bowel movements (*p* for trend = 0.019) and hard or lumpy stools (*p* for trend = 0.013). Compared with Q1, children in Q3 had significantly higher prevalences of infrequent bowel movements (aPR = 1.48, 95% CI: 1.09–2.00) and hard or lumpy stools (aPR = 1.23, 95% CI: 1.01–1.49), although the corresponding associations for Q4 did not reach statistical significance. These findings suggest that associations between UPF consumption and unfavorable dietary and health indicators may already be evident during early childhood, even in the absence of significant differences in anthropometric status.

The contribution of UPFs to total energy intake in this study (15.12%) was lower than that reported in several Western populations. However, comparisons with findings from other countries, including Asian populations, should be interpreted cautiously because of differences in food environments, dietary habits, age groups, dietary assessment methods, and UPF classification approaches. In particular, the present study adopted a modified NOVA-based classification to accommodate Taiwanese dietary characteristics. Nevertheless, accumulating evidence suggests that higher UPF consumption is generally associated with poorer overall dietary quality. Therefore, the nutritional implications of UPF consumption should be considered not only in terms of its contribution to total energy intake but also in relation to overall dietary composition and the potential displacement of nutrient-dense foods.

A major finding of the present study was the generally lower nutrient density observed among children with higher UPF intake. Specifically, higher UPF consumption was associated with lower intakes per 1000 kcal of dietary fiber, protein, potassium, calcium, magnesium, zinc, phosphorus, retinol equivalents, vitamins B1, B2, B6, B12, and vitamin C, together with higher densities of total fat, saturated fatty acids, and sodium. These findings are consistent with previous observations among Chilean preschool children, in whom greater UPF consumption was associated with lower intakes of important nutrients, including dietary fiber, protein, zinc, and vitamin A, and higher intakes of nutrients associated with non-communicable disease risk [23]. Together, these findings suggest that greater reliance on UPFs during early childhood may be accompanied by an unfavorable dietary nutrient profile, potentially reflecting the displacement of minimally processed, nutrient-dense foods.

The association between UPF intake and poorer nutrient profiles may be explained by two complementary mechanisms. First, many UPFs are formulated with refined starches, added sugars, unhealthy fats, and sodium while containing relatively limited amounts of fiber and micronutrients. Second, increasing reliance on UPFs may displace minimally processed foods, including fruits, vegetables, legumes, and traditional staple foods, which are important sources of essential nutrients. In the present study, higher UPF intake was accompanied by lower fiber density, which may have implications not only for nutrient adequacy but also for gastrointestinal function. Thus, UPF consumption may represent a broader dietary pattern characterized by reduced nutritional quality rather than simply the intake of individual processed products.

Despite the significant differences in dietary quality, no significant differences in height percentile, weight percentile, or BMI categories were observed across UPF intake quartiles in the present study. One possible explanation is that, during the preschool period, children have relatively limited total food intake; therefore, increased UPF consumption may primarily displace nutrient-dense foods rather than substantially increase overall energy intake. Although children in the highest UPF intake quartile had slightly higher energy intake than those in the lower quartiles, this difference may not have been sufficient to result in detectable changes in anthropometric indicators at this early developmental stage.

However, the absence of an association between UPF intake and anthropometric status should be interpreted cautiously. Previous longitudinal studies have suggested that the adverse effects of UPF consumption on adiposity may emerge later in life. In a prospective birth cohort study, higher UPF intake during childhood was associated with accelerated increases in BMI, fat mass index, body weight, and waist circumference from childhood to early adulthood [24]. Similarly, a longitudinal study among preschool children showed that higher UPF consumption during early childhood predicted greater increases in waist circumference during subsequent childhood [25]. Therefore, early exposure to a UPF-rich dietary pattern may have long-term implications for obesity risk, and continued longitudinal follow-up is warranted to determine whether higher UPF consumption during preschool years contributes to later adiposity development.

Dental caries was one of the strongest health outcomes associated with UPF intake in the present study. Dental caries prevalence increased progressively across UPF intake quartiles, from 12.8% in Q1 to 31.2% in Q4. After adjustment for age, sex, total energy intake, and home cooking frequency, children in the highest UPF intake quartile had a more than two-fold higher prevalence of dental caries compared with those in the lowest quartile (aPR = 2.270, 95% CI: 1.480–3.480; *p* < 0.001). These findings are consistent with previous evidence indicating that greater consumption of UPFs is associated with increased risk of dental caries among children and adolescents. A systematic review and meta-analysis by Cascaes et al. reported a positive association between UPF consumption and dental caries, suggesting that diets characterized by a high proportion of industrially processed foods may contribute to unfavorable oral health outcomes in younger populations [16]. Similarly, de Souza et al. demonstrated that higher consumption of ultra-processed foods was associated with early childhood caries among Brazilian children, supporting the relevance of UPF exposure during the preschool period [17].

The relationship between UPF intake and dental caries may not be explained solely by sugar content. Although free sugars are well-established contributors to cariogenesis, UPFs represent a broader dietary pattern that may involve multiple behavioral and nutritional pathways. UPF-rich diets are often characterized by frequent consumption of snacks, repeated exposure to refined carbohydrates, prolonged contact of fermentable carbohydrates with tooth surfaces, and unfavorable food textures that may influence oral clearance. In addition, increased reliance on UPFs may reflect an eating pattern involving frequent between-meal snacking and displacement of minimally processed foods that contribute to a healthier dietary profile. Therefore, UPF intake may capture the cumulative influence of food composition, eating frequency, and dietary behaviors beyond the effect of individual nutrients.

The present findings are also supported by previous evidence from Taiwanese preschool children. Yen et al. reported that dietary intake patterns were associated with dental caries among preschool children in Taiwan, with higher vegetable intake being associated with lower caries scores and frequent between-meal snacking associated with greater caries risk [26]. Consistently, in the present study, higher vegetable intake was independently associated with a lower prevalence of dental caries (aPR = 0.810, 95% CI: 0.660–0.990; *p* = 0.038) after mutual adjustment for other food groups. Although vegetables are unlikely to exert a direct protective effect against dental caries, greater vegetable consumption may represent an overall dietary pattern characterized by higher intake of minimally processed foods and lower reliance on cariogenic snack foods.

Constipation-related symptoms represent another potential health outcome associated with UPF consumption. In the present study, higher UPF intake was associated with a greater prevalence of caregiver-reported infrequent bowel movements and hard or lumpy stools. After adjustment for age group, sex, and frequency of home cooking, children in the third UPF intake quartile (Q3) had significantly higher prevalences of infrequent bowel movements (aPR = 1.48, 95% CI: 1.09–2.00; *p* = 0.012) and hard or lumpy stools (aPR = 1.23, 95% CI: 1.01–1.49; *p* = 0.037) compared with those in Q1. Significant positive trends were also observed across UPF intake quartiles for both symptoms (*p* for trend = 0.019 and 0.013, respectively), although the associations for Q4 did not reach statistical significance. These findings suggest that higher UPF consumption may be associated with unfavorable bowel habits among preschool children, although the associations were not strictly monotonic across quartiles.

Direct evidence regarding the association between UPF intake and constipation-related symptoms among young children remains limited, as most epidemiological studies have focused on adults. In a cross-sectional analysis of 12716 U.S. adults using NHANES data, Lo et al. reported that participants in the highest UPF intake quartile had more than twice the odds of constipation compared with those in the lowest quartile (adjusted OR = 2.20, 95% CI: 1.76–2.74). This association remained significant after adjustment for dietary quality, fiber, and water intake, suggesting that dietary characteristics associated with UPF consumption may contribute to differences in bowel habits beyond conventional nutritional factors [27]. Although these findings were derived from adults and cannot be directly extrapolated to preschool children, they provide additional epidemiological evidence supporting further investigation of the relationship between UPF consumption and gastrointestinal function during early childhood.

Several dietary mechanisms may contribute to the observed associations between UPF consumption and bowel-related symptoms. UPF-rich dietary patterns are often characterized by lower dietary fiber density and reduced consumption of minimally processed, nutrient-dense foods. Consistent with this possibility, dietary fiber density in the present study decreased progressively across increasing UPF intake quartiles, from 8.4 g/1000 kcal in Q1 to 5.9 g/1000 kcal in Q4. Dietary fiber contributes to normal bowel function by increasing stool bulk, enhancing water retention, and supporting intestinal motility, partly through microbial fermentation. Furthermore, differences in food structure, water content, and additives between UPFs and minimally processed foods may also influence gastrointestinal function. However, these potential mechanisms were not directly investigated in the present study, and the extent to which lower dietary fiber intake explains the observed associations remains uncertain. A systematic review involving children aged 0–5 years suggested that dietary fiber may influence bowel function during early childhood, although the available evidence remains limited and heterogeneous [28].

The food group-specific analyses provided additional insights into the associations between dietary intake and individual bowel-related symptoms. Higher fruit intake was associated with lower odds of reporting more frequent episodes of infrequent bowel movements (aOR = 0.872, 95% CI: 0.801–0.948; *p* = 0.0014), whereas higher biscuit/snack intake was associated with greater odds of reporting more frequent episodes of hard or lumpy stools (aOR = 1.324, 95% CI: 1.073–1.634; *p* = 0.0090). Fruits provide dietary fiber, water, and bioactive compounds that may contribute to normal bowel function. In contrast, biscuits and packaged snacks often contain refined carbohydrates and fats while providing relatively limited amounts of dietary fiber. These findings suggest that different food groups may be associated with distinct aspects of bowel habits among preschool children. Nevertheless, these exploratory associations should be interpreted cautiously, as the study design does not establish causal relationships.

These findings are broadly consistent with previous studies examining dietary factors and bowel habits among young children. Park et al. reported that lower consumption of fruits and vegetables was associated with functional constipation among children attending daycare centers [29]. Similarly, Asakura et al. identified associations between dietary intake patterns and constipation among Japanese preschool children [30]. Together with the available evidence regarding dietary fiber and bowel function [28], these observations suggest that overall dietary patterns, rather than individual nutrients alone, may be relevant to bowel health during early childhood. However, because the present study assessed caregiver-reported bowel-related symptoms rather than clinically diagnosed functional constipation, direct comparisons with studies using clinical diagnostic criteria should be interpreted cautiously. Further prospective studies incorporating validated pediatric bowel assessments are needed to clarify the relationships between UPF consumption, dietary patterns, and gastrointestinal health during early childhood.

This study has several strengths. It examined multiple nutritional and health indicators in 822 preschool children recruited through a kindergarten-based health screening program. Anthropometric and dental assessments were performed by trained healthcare professionals rather than relying solely on parental reports. Together, these approaches provide a broad descriptive assessment of associations between UPF intake and health indicators during early childhood.

Several limitations should be acknowledged. First, the cross-sectional design precludes establishing temporal relationships or causal associations between UPF intake and the examined health outcomes. Reverse causation cannot be excluded, as dental problems or gastrointestinal symptoms may influence children’s food preferences and caregivers’ dietary choices. Moreover, dental caries experience reflects accumulated disease history, whereas dietary intake was assessed over the preceding month, further limiting temporal interpretation. The study was conducted among preschool children attending kindergartens involved in a health examination program in a single district of Taipei. Although all eligible kindergartens within the program were invited, participation at the individual child level was voluntary. The relatively low questionnaire participation rate may have introduced selection bias, limiting the generalizability of the findings. In addition, anthropometric and dental examination data were unavailable for some participants, potentially introducing further selection bias.

Second, dietary intake was assessed using a caregiver-reported semi-quantitative food frequency questionnaire (SQ-FFQ), which may be subject to recall bias, social-desirability bias, and errors in estimating portion sizes. Although the questionnaire was developed by the research team, reviewed by two experts in pediatric nutrition and child health, and finalized through an expert meeting, it had not undergone formal reliability and validity testing in Taiwanese preschool children. Furthermore, the adapted NOVA-based classification differed from the original framework for certain food categories, and incomplete information on food ingredients, formulations, and processing methods may have resulted in exposure misclassification. Although foods were independently classified by two nutrition researchers and disagreements were resolved through consensus, quantitative inter-rater agreement was not assessed. Additionally, the available dietary data did not allow added sugars to be reliably distinguished from naturally occurring sugars, preventing direct assessment of the association between added sugar intake and dental caries.

Third, dental caries was assessed through routine clinical examinations based on dentists’ professional judgment rather than a formally standardized diagnostic scoring system, potentially introducing measurement variability. Although decayed and filled primary and permanent teeth were recorded separately, they were combined to assess overall caries experience. Differences in tooth eruption and exposure duration across the study age range may have affected the comparability of dental outcomes. Furthermore, important potential confounding factors, including toothbrushing practices, fluoride exposure, dental care utilization, parental education, socioeconomic status, and the frequency of consumption of cariogenic foods, were not comprehensively assessed. Therefore, residual confounding remains an important consideration when interpreting the association between UPF intake and dental caries.

Finally, bowel symptoms were reported by caregivers rather than assessed through direct clinical evaluation. The questionnaire did not fully incorporate the age-specific pediatric Rome IV diagnostic criteria, and some subjective symptoms may be difficult for caregivers to evaluate reliably in young children. Therefore, these findings represent associations with individual constipation-related symptoms rather than clinically diagnosed functional constipation. The three-month bowel-symptom assessment period also differed from the one-month dietary assessment period, which may have introduced additional measurement variability. Moreover, the proportional odds assumption was not satisfied for the ordinal regression model examining infrequent bowel movements, requiring cautious interpretation of its common adjusted odds ratios. Multiple exploratory food-group comparisons may also increase the likelihood of chance findings.

Future prospective studies incorporating validated dietary and bowel assessment tools, standardized pediatric dental examinations, more comprehensive confounder measurements, and longitudinal follow-up are needed to confirm these findings and clarify the potential relationships between UPF consumption and health outcomes during early childhood.

## 5. Conclusions

In conclusion, higher ultra-processed food (UPF) intake among Taiwanese preschool children was associated with an unfavorable dietary nutrient profile, characterized by lower intakes of dietary fiber, protein, and several essential micronutrients, together with higher intakes of saturated fatty acids and sodium. Although no significant differences in anthropometric status were observed across UPF intake quartiles, higher UPF consumption was associated with a greater prevalence of dental caries. Furthermore, significant positive trends were observed between UPF intake quartiles and the prevalence of caregiver-reported infrequent bowel movements and hard or lumpy stools, although associations for individual quartiles were not consistently significant. Exploratory food-group analyses further suggested that higher fruit intake was associated with less frequent reports of infrequent bowel movements, whereas higher biscuit/snack intake was associated with more frequent reports of hard or lumpy stools.

These findings highlight the potential relevance of UPF consumption to nutritional quality, oral health, and bowel-related symptoms during early childhood. However, given the cross-sectional design and potential residual confounding, the observed associations should not be interpreted as causal relationships. Dietary guidance for preschool children should continue to emphasize balanced diets rich in minimally processed, nutrient-dense foods, including fruits, vegetables, and other fiber-rich foods. Further longitudinal studies incorporating validated dietary assessments and comprehensive pediatric health evaluations are warranted to clarify the long-term relationships between UPF consumption and children’s nutritional and health outcomes.

## Figures and Tables

**Table 1:** Basic Characteristics of Preschool Children According to Quartiles of Ultra-Processed Food Intake.

Characteristics	Total (*n* = 822)	Q1 (*n* = 207)	Q2 (*n* = 205)	Q3 (*n* = 205)	Q4 (*n* = 205)	*p* Value
Sex						0.094
Male	440 (53.5)	123 (59.4)	114 (55.6)	105 (51.2)	98 (47.8)	
Female	382 (46.5)	84 (40.6)	91 (44.4)	100 (48.8)	107 (52.2)	
Age						0.154
2 ≤ age < 4 years	176 (21.4)	59 (28.5)	41 (20.0)	43 (21.0)	33 (16.1)	
4 ≤ age < 5 years	217 (26.4)	52 (25.1)	52 (25.4)	55 (26.8)	58 (28.3)	
5 ≤ age < 6 years	239 (29.1)	59 (28.5)	62 (30.2)	53 (25.9)	65 (31.7)	
6 ≤ age < 7 years	190 (23.1)	37 (17.9)	50 (24.4)	54 (26.3)	49 (23.9)	
Home Food Preparation and Convenience Food Consumption						
Frequency of home cooking (times/week)	6.7 ± 5.8	6.8 ± 5.9	6.7 ± 5.7	6.8 ± 5.8	6.4 ± 5.8	0.870
Frequency of purchasing refrigerated ready-to-eat foods for the child (times/week)	0.8 ± 1.8	0.5 ± 1.4 ^a^	0.6 ± 1.0 ^a^	0.9 ± 2.0 ^a^^b^	1.3 ± 2.5 ^b^	<0.001 *
Frequency of purchasing frozen foods for the child (times/week)	0.8 ± 1.9	0.4 ± 0.8 ^a^	0.6 ± 1.1 ^a^	0.9 ± 2.0 ^a^^b^	1.3 ± 2.9 ^b^	<0.001 *

Values are presented as mean ± standard deviation or number (percentage). Q1–Q4 indicate quartiles of the percentage of total energy intake derived from ultra-processed foods (UPFs): Q1, ≤7.8%; Q2, >7.8% to ≤13.1%; Q3, >13.1% to ≤20.4%; and Q4, >20.4%. Continuous variables were compared among groups using one-way analysis of variance, followed by Scheffé’s post hoc test when the overall *p* value was statistically significant. Values in the same row with different superscript letters indicate significant differences between groups (*p* < 0.05), whereas values with the same superscript letters indicate no significant difference. Categorical variables were presented as number (percentage) and analyzed using the chi-square test. A two-sided *p* value < 0.05 was considered statistically significant. * Indicates a statistically significant overall difference among quartiles based on one-way analysis of variance (*p* < 0.05).

**Table 2:** Energy and Nutrient Intakes across Quartiles of the Percentage of Total Energy Intake Derived from Ultra-Processed Foods among Preschool Children.

Nutrients	Q1 (*n* = 207)	Q2 (*n* = 205)	Q3 (*n* = 205)	Q4 (*n* = 205)	*p* Value
Energy intake (kcal/day)	1516 ± 565 ^a^	1470 ± 609 ^a^	1443 ± 616 ^a^	1634 ± 1069 ^a^	0.049 *
Macronutrients, per 1000 kcal					
Carbohydrate (g)	150 ± 21 ^a^	152 ± 18 ^a^	148 ± 18 ^a^	147 ± 16 ^a^	0.022 *
Dietary fiber (g)	8.4 ± 3.2 ^a^	7.6 ± 2.7 ^b^	7.1 ± 2.6 ^b^	5.9 ± 2.3 ^c^	<0.001 *
Protein (g)	41 ± 6 ^a^	38 ± 6 ^b^	38 ± 6 ^b^	35 ± 5 ^c^	<0.001 *
Total fat (g)	28 ± 7 ^a^	28 ± 6 ^a^	30 ± 6 ^b^	32 ± 5 ^b^	<0.001 *
Saturated fatty acids (g)	11 ± 4 ^a^	11 ± 3 ^a^^b^	12 ± 3 ^b^	13 ± 3 ^c^	<0.001 *
Minerals and Vitamins, per 1000 kcal					
Sodium (mg)	657 ± 199 ^a^	689 ± 197 ^a^	753 ± 194 ^b^	849 ± 216 ^c^	<0.001 *
Potassium (mg)	1575 ± 441 ^a^	1401 ± 416 ^b^	1327 ± 381 ^b^	1127 ± 333 ^c^	<0.001 *
Calcium (mg)	462 ± 199 ^a^	405 ± 193 ^b^	373 ± 148 ^b^^c^	337 ± 145 ^c^	<0.001 *
Magnesium (mg)	151 ± 36 ^a^	136 ± 32 ^b^	128 ± 32 ^b^	109 ± 25 ^c^	<0.001 *
Iron (mg)	5.8 ± 1.7	5.4 ± 1.5	5.6 ± 1.7	5.4 ± 1.8	0.093
Zinc (mg)	6.1 ± 0.9 ^a^	5.6 ± 0.9 ^b^	5.3 ± 1.0 ^b^	4.7 ± 1.1 ^c^	<0.001 *
Phosphorus (mg)	631 ± 150 ^a^	579 ± 142 ^b^	544 ± 127 ^b^	488 ± 114 ^c^	<0.001 *
Retinol equivalents (RE) (µg)	742 ± 327 ^a^	624 ± 278 ^b^	571 ± 261 ^b^	460 ± 282 ^c^	<0.001 *
α-Tocopherol equivalents (α-TE) (mg)	4.2 ± 1.4	4.0 ± 1.3	4.3 ± 1.7	4.0 ± 1.7	0.113
Vitamin B1 (mg)	0.65 ± 0.14 ^a^	0.60 ± 0.12 ^b^	0.59 ± 0.13 ^b^	0.53 ± 0.12 ^c^	<0.001 *
Vitamin B2 (mg)	0.97 ± 0.32 ^a^	0.89 ± 0.32 ^a^^b^	0.85 ± 0.27 ^b^^c^	0.77 ± 0.27 ^c^	<0.001 *
Niacin (mg)	6.9 ± 1.8	6.7 ± 1.7	6.8 ± 1.9	6.6 ± 1.7	0.196
Vitamin B6 (mg)	1.0 ± 0.3 ^a^	0.9 ± 0.3 ^b^	0.9 ± 0.3 ^b^	0.7 ± 0.3 ^c^	<0.001 *
Vitamin B12 (µg)	3.4 ± 2.6 ^a^	2.9 ± 1.7 ^a^^b^	3.1 ± 1.9 ^a^^b^	2.8 ± 2.1 ^b^	0.009 *
Vitamin C (mg)	78 ± 41^a^	68 ± 34 ^b^	68 ± 33 ^b^	57 ± 29 ^c^	<0.001 *

Values are presented as mean ± standard deviation. Q1–Q4 indicate quartiles of the percentage of total energy intake derived from ultra-processed foods (UPFs): Q1, ≤7.8%; Q2, >7.8% to ≤13.1%; Q3, >13.1% to ≤20.4%; and Q4, >20.4%. Energy intake is presented as kcal/day, whereas macronutrients, minerals, and vitamins are presented per 1000 kcal. Differences across quartiles were examined using one-way analysis of variance, followed by Scheffé’s post hoc test for multiple comparisons when the overall *p* value was statistically significant. Values in the same row with different superscript letters indicate significant differences between groups (*p* < 0.05), whereas values with the same superscript letters indicate no significant difference. A two-sided *p* value < 0.05 was considered statistically significant. * Indicates a statistically significant overall difference among quartiles based on one-way analysis of variance (*p* < 0.05). RE, retinol equivalents; α-TE, α-tocopherol equivalents.

**Table 3:** Height and Weight Percentiles and Anthropometric Status across Quartiles of the Percentage of Total Energy Intake Derived from Ultra-Processed Foods among Preschool Children.

**Variables**	**Total** **(** ***n* = 777)**	**Q1** **(** ***n* = 197)**	**Q2** **(** ***n* = 196)**	**Q3** **(** ***n* = 193)**	**Q4** **(** ***n* = 191)**	***p* Value**
Height percentile	37 ± 28	36 ± 29	38 ± 27	35 ± 27	40 ± 30	0.290
Weight percentile	46 ± 28	46 ± 27	46 ± 27	45 ± 29	47 ± 30	0.829
Anthropometric status						0.858
Underweight	20 (2.6)	7 (3.6)	5 (2.6)	2 (1.0)	6 (3.1)	
Normal weight	610 (78.5)	148 (75.1)	157 (80.1)	157 (81.3)	148 (77.5)	
Overweight	90 (11.6)	25 (12.7)	20 (10.2)	21 (10.9)	24 (12.6)	
Obesity	57 (7.3)	17 (8.6)	14 (7.1)	13 (6.7)	13 (6.8)	

Values are presented as mean ± standard deviation (SD) for continuous variables and number (percentage) [*n* (%)] for categorical variables. Q1–Q4 indicate quartiles of the percentage of total energy intake derived from ultra-processed foods (UPFs): Q1, ≤7.8%; Q2, >7.8% to ≤13.1%; Q3, >13.1% to ≤20.4%; and Q4, >20.4%. Height and weight percentiles were determined using sex- and age-specific Taiwanese child growth reference values. Anthropometric status was categorized as underweight, normal weight, overweight, or obesity according to the body mass index (BMI) reference standards established by the Health Promotion Administration, Ministry of Health and Welfare, Taiwan. Differences in continuous variables across UPF intake quartiles were assessed using one-way analysis of variance (ANOVA), whereas categorical variables were compared using Pearson’s chi-square test. All statistical tests were two-sided, and *p* < 0.05 was considered statistically significant.

**Table 4:** Dental Caries Status and Number of Filled and Unfilled Decayed Teeth across Quartiles of the Percentage of Total Energy Intake Derived from Ultra-Processed Foods among Preschool Children.

Variables	Total (*n* = 746)	Q1 (*n* = 188)	Q2 (*n* = 188)	Q3 (*n* = 184)	Q4 (*n* = 186)	*p* Value
Dental caries status						<0.001 *
No, *n* (%)	578 (77.5)	164 (87.2)	149 (79.3)	137 (74.5)	128 (68.8)	
Yes, *n* (%)	168 (22.5)	24 (12.8)	39 (20.7)	47 (25.5)	58 (31.2)	
Number of caries-affected teeth	0.7 ± 1.7	0.3 ± 1.0 ^a^	0.6 ± 1.6 ^a^^b^	0.8 ± 1.8 ^a^^b^	1.1 ± 2.2 ^b^	<0.001 *

Values are presented as mean ± standard deviation or number (percentage). Q1–Q4 indicate quartiles of the percentage of total energy intake derived from ultra-processed foods (UPFs): Q1, ≤7.8%; Q2, >7.8% to ≤13.1%; Q3, >13.1% to ≤20.4%; and Q4, >20.4%. The number of dental caries was defined as the total number of affected teeth, calculated by summing decayed primary teeth, decayed permanent teeth, filled primary teeth, and filled permanent teeth. Dental caries status was analyzed using the chi-square test. The number of caries-affected teeth was compared across quartiles using one-way analysis of variance, followed by Scheffé’s post hoc test for multiple comparisons when the overall *p* value was statistically significant. Values in the same row with different superscript letters indicate significant differences between groups (*p* < 0.05), whereas values with the same superscript letters indicate no significant difference. A two-sided *p* value < 0.05 was considered statistically significant. * Indicates a statistically significant overall difference among quartiles based on one-way analysis of variance (*p* < 0.05).

**Table 5:** Distribution of caregiver-reported infrequent bowel movements and hard stool frequency across quartiles of ultra-processed food intake among preschool children.

Symptom/Frequency Score	Q1 (*n* = 207)	Q2 (*n* = 205)	Q3 (*n* = 205)	Q4 (*n* = 205)	MC *p*	FDR *p*
Infrequent bowel movements < 3 times/week						
0—Never or rarely	157 (75.8%)	146 (71.2%)	131 (63.9%)	138 (67.3%)	0.430	0.516
1—Sometimes	16 (7.7%)	19 (9.3%)	24 (11.7%)	19 (9.3%)		
2—Often	7 (3.4%)	9 (4.4%)	9 (4.4%)	11 (5.4%)		
3—Most of the time	6 (2.9%)	6 (2.9%)	4 (2.0%)	9 (4.4%)		
4—Almost always	21 (10.1%)	25 (12.2%)	37 (18.0%)	28 (13.7%)		
Hard/lumpy stools						
0—Never or rarely	112 (54.1%)	114 (55.6%)	92 (44.9%)	95 (46.3%)	0.004	0.026
1—Sometimes	73 (35.3%)	71 (34.6%)	91 (44.4%)	68 (33.2%)		
2—Often	9 (4.3%)	11 (5.4%)	9 (4.4%)	27 (13.2%)		
3—Most of the time	9 (4.3%)	5 (2.4%)	5 (2.4%)	8 (3.9%)		
4—Almost always	4 (1.9%)	4 (2.0%)	8 (3.9%)	7 (3.4%)		

Values are presented as numbers and percentages [*n* (%)] within each UPF intake quartile. Q1–Q4 indicate quartiles of the percentage of total energy intake derived from ultra-processed foods (UPFs): Q1, ≤7.8%; Q2, >7.8% to ≤13.1%; Q3, >13.1% to ≤20.4%; and Q4, >20.4%. Symptom frequency was assessed using a five-point ordinal scale: 0 = never or rarely, 1 = sometimes, 2 = often, 3 = most of the time, and 4 = almost always. MC *p* represents the fixed-margin Monte Carlo *p*-value for comparisons of the five-level symptom distributions across UPF quartiles, based on 100,000 simulated contingency tables. FDR *p* represents the Benjamini–Hochberg false discovery rate-adjusted *p*-value, accounting for multiple comparisons across all six constipation-related symptoms.

**Table 6:** Multivariable Association between Ultra-Processed Food Intake and Dental Caries Prevalence among Preschool Children.

UPF Intake Quartile	Adjusted Prevalence Ratio (aPR) (95% CI)	*p* Value
Q1	1.000 (reference)	—
Q2	1.580 (0.997–2.500)	0.052
Q3	1.960 (1.250–3.050)	0.003
Q4	2.270 (1.480–3.480)	<0.001
*p* for trend	—	<0.001

Values are presented as adjusted prevalence ratios (aPRs) with 95% confidence intervals (CIs). Q1–Q4 indicate quartiles of the percentage of total energy intake derived from ultra-processed foods (UPFs): Q1, ≤7.8%; Q2, >7.8% to ≤13.1%; Q3, >13.1% to ≤20.4%; and Q4, >20.4%. Dental caries prevalence was defined as the presence of at least one decayed or filled tooth. Modified Poisson regression with robust standard errors was used to estimate aPRs, with Q1 as the reference group. The model was adjusted for age group, sex, total energy intake, and frequency of home cooking. The *p* value for trend was obtained by entering UPF intake quartiles as an ordinal variable in the regression model. A two-sided *p* value < 0.05 was considered statistically significant.

**Table 7:** Adjusted prevalence ratios for caregiver-reported infrequent bowel movements and hard stool symptoms across UPF intake quartiles.

Outcome	UPF Quartile	With Symptom *n* (%)	aPR (95% CI)	*p* Value	*p* for Trend
Infrequent bowel movements (<3/week)	Q1	50 (24.2%)	1.000 (reference)	—	0.019
	Q2	59 (28.8%)	1.170 (0.840–1.610)	0.350	
	Q3	74 (36.1%)	1.480 (1.090–2.000)	0.012	
	Q4	67 (32.7%)	1.350 (0.990–1.830)	0.058	
Hard/lumpy stools	Q1	95 (45.9%)	1.000 (reference)	—	0.013
	Q2	91 (44.4%)	0.990 (0.800–1.230)	0.947	
	Q3	113 (55.1%)	1.230 (1.010–1.490)	0.037	
	Q4	110 (53.7%)	1.213 (0.995–1.477)	0.056	

Values are presented as adjusted prevalence ratios (aPRs) with 95% confidence intervals (CIs). Q1–Q4 indicate quartiles of the percentage of total energy intake derived from ultra-processed foods (UPFs): Q1, ≤7.8%; Q2, >7.8% to ≤13.1%; Q3, >13.1% to ≤20.4%; and Q4, >20.4%. Symptoms were dichotomized as 0 (never or rarely) versus 1–4 (at least sometimes). Modified Poisson regression with HC3 robust standard errors was performed, adjusting for age group, sex, and frequency of home cooking, with Q1 as the reference group. Linear trends were assessed by entering UPF quartiles as an ordinal variable in the regression models. All *p* values were two-sided, and *p* < 0.05 was considered statistically significant.

**Table 8:** Associations of Daily Food Group Intakes with Caregiver-Reported Infrequent Bowel Movements and Hard Stools among Preschool Children.

Food Group (Servings/Day)	Infrequent Bowel Movements		Hard Stools	
	aOR (95% CI)	*p* Value	aOR (95% CI)	*p* Value
Whole grains	0.923 (0.801–1.063)	0.2667	1.072 (0.951–1.208)	0.2560
Vegetables	0.997 (0.896–1.109)	0.9534	1.010 (0.923–1.106)	0.8258
Fruit	0.872 (0.801–0.948)	0.0014	0.998 (0.934–1.067)	0.9581
Legumes	1.168 (0.554–2.462)	0.6835	0.479 (0.217–1.056)	0.0681
Dairy products	1.051 (0.958–1.154)	0.2893	1.040 (0.955–1.132)	0.3697
Biscuits/snacks	1.149 (0.901–1.464)	0.2629	1.324 (1.073–1.634)	0.0090
Sugary foods/beverages	0.935 (0.799–1.093)	0.3981	1.077 (0.937–1.238)	0.2964

Values are presented as adjusted odds ratios (aORs) with 95% confidence intervals (CIs). Associations were analyzed using multivariable ordinal logistic regression. Infrequent bowel movements and hard stools were assessed using caregiver-reported five-point frequency scales: 0 = never or rarely, 1 = sometimes, 2 = often, 3 = most of the time, and 4 = almost always. All seven food groups were entered simultaneously into the regression models, with adjustment for age, sex, and total energy intake. The aOR represents the odds of reporting a higher symptom-frequency category per unit increase in daily food group intake, while holding other variables constant. All *p* values were two-sided, and *p* < 0.05 was considered statistically significant.

**Table 9:** Multivariable Associations of Daily Food Group Intakes with Dental Caries among Preschool Children.

Food Group, Servings/Day	Dental Caries Prevalence, aPR (95% CI)	*p* Value	Caries Count, aIRR (95% CI)	*p* Value
Whole grains	1.10 (0.93–1.31)	0.256	1.12 (0.90–1.38)	0.323
Vegetables	0.81 (0.66–0.99)	0.038 *	0.85 (0.69–1.04)	0.116
Fruit	0.93 (0.83–1.05)	0.253	0.88 (0.76–1.02)	0.099
Legumes	1.33 (0.41–4.30)	0.632	0.43 (0.10–1.80)	0.247
Dairy products	0.88 (0.74–1.04)	0.127	0.96 (0.80–1.16)	0.682
Biscuits/snacks	1.28 (0.88–1.87)	0.192	1.49 (0.83–2.69)	0.180
Sugary foods/beverages	1.15 (0.93–1.43)	0.193	1.54 (0.99–2.38)	0.054

Values are presented as adjusted prevalence ratios (aPRs) or adjusted incidence rate ratios (aIRRs) with 95% confidence intervals (CIs). All food groups were entered simultaneously into the multivariable models, with adjustment for age, sex, and total energy intake. Dental caries prevalence (yes/no) was analyzed using modified Poisson regression with robust standard errors, whereas the number of caries-affected teeth was analyzed using negative binomial regression with robust standard errors. The caries count was calculated as the sum of decayed and filled primary and permanent teeth. Effect estimates represent the relative change in dental caries prevalence or expected caries count associated with a one-serving/day increase in the corresponding food group, while holding the other food groups and covariates constant. Sugary snacks and sugar-sweetened beverages were combined into a single “sugary foods/beverages” variable because of substantial multicollinearity when modeled separately. Following this combination, all variance inflation factors were below 2 (range, 1.04–1.19), indicating no problematic multicollinearity. A two-sided *p* value < 0.05 was considered statistically significant. * Indicates a statistically significant association (*p* < 0.05).

## Data Availability

The datasets used and analyzed during the current study are available from the corresponding author on reasonable request.

## Supplementary Materials

The following supporting information can be downloaded at https://www.mdpi.com/article/10.3390/nu18213670/s1. Table S1: Full distribution of six caregiver-reported bowel symptoms across quartiles of ultra-processed food intake among preschool children; Table S2: Adjusted prevalence ratios for all six dichotomized caregiver-reported symptoms across UPF intake quartiles.

## Abbreviations

ANOVA	analysis of variance
aIRR	adjusted incidence rate ratio
aOR	adjusted odds ratio
aPR	adjusted prevalence ratio
BMI	body mass index
CI	confidence interval
FDR	false discovery rate
FFQ	food frequency questionnaire
HC3	heteroskedasticity-consistent standard errors (type 3)
IRB	Institutional Review Board
MC	Monte Carlo
Q1–Q4	first through fourth quartiles of UPF intake
RE	retinol equivalents
SD	standard deviation
SQ-FFQ	semi-quantitative food frequency questionnaire
UPF	ultra-processed food
VIF	variance inflation factor
α-TE	α-tocopherol equivalents
