# Supplementary material for: Ultra-Processed Food Intake Is Associated with Dietary Quality, Dental Caries, and Constipation-Related Symptoms Among Preschool Children in Taiwan: A Cross-Sectional Study
Source: Nutrients. 2026 Nov 5;18(21):3670. doi: 10.3390/nu18213670 (PMC13765253; doi:10.3390/nu18213670)
Supplement: Supplementary file 1 [file nutrients-18-03670-s001.zip › nutrients-4600959-supplementary.pdf]

Supplementary Table S1. Full distribution of six caregiver-reported bowel symptoms across quartiles of ultra-processed food intake among preschool children.

| Symptom / Frequency score | Q1 (*n*=207) | Q2 (*n*=205) | Q3 (*n*=205) | Q4 (*n*=205) | MC p | FDR p |
|---|---|---|---|---|---|---|
| Infrequent bowel movements (<3/week) | | | | | | |
| 0 — Never/rarely | 157 (75.8%) | 146 (71.2%) | 131 (63.9%) | 138 (67.3%) | 0.430 | 0.516 |
| 1 — Sometimes | 16 (7.7%) | 19 (9.3%) | 24 (11.7%) | 19 (9.3%) | | |
| 2 — Often | 7 (3.4%) | 9 (4.4%) | 9 (4.4%) | 11 (5.4%) | | |
| 3 — Most of the time | 6 (2.9%) | 6 (2.9%) | 4 (2.0%) | 9 (4.4%) | | |
| 4 — Almost always | 21 (10.1%) | 25 (12.2%) | 37 (18.0%) | 28 (13.7%) | | |
| Straining during defecation | | | | | | |
| 0 — Never/rarely | 144 (69.6%) | 131 (63.9%) | 141 (68.8%) | 131 (63.9%) | 0.645 | 0.645 |
| 1 — Sometimes | 53 (25.6%) | 59 (28.8%) | 57 (27.8%) | 65 (31.7%) | | |
| 2 — Often | 8 (3.9%) | 9 (4.4%) | 4 (2.0%) | 6 (2.9%) | | |
| 3 — Most of the time | 1 (0.5%) | 3 (1.5%) | 3 (1.5%) | 1 (0.5%) | | |
| 4 — Almost always | 1 (0.5%) | 3 (1.5%) | 0 (0.0%) | 2 (1.0%) | | |
| Hard/lumpy stools | | | | | | |
| 0 — Never/rarely | 112 (54.1%) | 114 (55.6%) | 92 (44.9%) | 95 (46.3%) | 0.004 | 0.026 |
| 1 — Sometimes | 73 (35.3%) | 71 (34.6%) | 91 (44.4%) | 68 (33.2%) | | |
| 2 — Often | 9 (4.3%) | 11 (5.4%) | 9 (4.4%) | 27 (13.2%) | | |
| 3 — Most of the time | 9 (4.3%) | 5 (2.4%) | 5 (2.4%) | 8 (3.9%) | | |
| 4 — Almost always | 4 (1.9%) | 4 (2.0%) | 8 (3.9%) | 7 (3.4%) | | |
| Sensation of incomplete evacuation | | | | | | |
| 0 — Never/rarely | 168 (81.2%) | 156 (76.1%) | 152 (74.1%) | 144 (70.2%) | 0.192 | 0.400 |
| 1 — Sometimes | 37 (17.9%) | 43 (21.0%) | 46 (22.4%) | 53 (25.9%) | | |
| 2 — Often | 1 (0.5%) | 2 (1.0%) | 5 (2.4%) | 4 (2.0%) | | |
| 3 — Most of the time | 1 (0.5%) | 1 (0.5%) | 2 (1.0%) | 3 (1.5%) | | |
| 4 — Almost always | 0 (0.0%) | 3 (1.5%) | 0 (0.0%) | 1 (0.5%) | | |
| Sensation of anorectal blockage | | | | | | |
| 0 — Never/rarely | 174 (84.1%) | 156 (76.1%) | 161 (78.5%) | 154 (75.1%) | 0.200 | 0.400 |
| 1 — Sometimes | 31 (15.0%) | 41 (20.0%) | 39 (19.0%) | 42 (20.5%) | | |
| 2 — Often | 2 (1.0%) | 6 (2.9%) | 5 (2.4%) | 5 (2.4%) | | |

| Symptom / Frequency score | Q1 (*n*=207) | Q2 (*n*=205) | Q3 (*n*=205) | Q4 (*n*=205) | MC p | FDR p |
|---|---|---|---|---|---|---|
| 3 — Most of the time | 0 (0.0%) | 0 (0.0%) | 0 (0.0%) | 2 (1.0%) | | |
| 4 — Almost always | 0 (0.0%) | 2 (1.0%) | 0 (0.0%) | 2 (1.0%) | | |
| Manual maneuvers to facilitate defecation | | | | | | |
| 0 — Never/rarely | 200 (96.6%) | 201 (98.0%) | 196 (95.6%) | 198 (96.6%) | 0.301 | 0.452 |
| 1 — Sometimes | 7 (3.4%) | 4 (2.0%) | 7 (3.4%) | 3 (1.5%) | | |
| 2 — Often | 0 (0.0%) | 0 (0.0%) | 1 (0.5%) | 3 (1.5%) | | |
| 3 — Most of the time | 0 (0.0%) | 0 (0.0%) | 0 (0.0%) | 0 (0.0%) | | |
| 4 — Almost always | 0 (0.0%) | 0 (0.0%) | 1 (0.5%) | 1 (0.5%) | | |

Reported values are within-quartile n (%). Analyses use 5 × 4 Pearson $\chi^2$ tests with fixed-margin Monte Carlo p values (100,000 draws). Manual-maneuver score 3 had zero observations in all quartiles and was excluded from the $\chi^2$ calculation (4 × 4, df = 9). The other five symptoms use df = 12. FDR adjustment includes six symptom-level tests.

Supplementary Table S2. Adjusted prevalence ratios for all six dichotomized caregiver-reported symptoms across UPF intake quartiles.

| **Outcome** | **UPF quartile** | **With symptom *n* (%)** | **aPR (95% CI)** | **p value** | **p for trend** |
|---|---|---|---|---|---|
| Infrequent bowel movements (<3/week) | Q1 | 50 (24.2%) | 1.00 (reference) | — | 0.019 |
| | Q2 | 59 (28.8%) | 1.17 (0.84–1.61) | 0.350 | |
| | Q3 | 74 (36.1%) | 1.48 (1.09–2.00) | 0.012 | |
| | Q4 | 67 (32.7%) | 1.35 (0.99–1.83) | 0.058 | |
| Straining during defecation | Q1 | 63 (30.4%) | 1.00 (reference) | — | 0.306 |
| | Q2 | 74 (36.1%) | 1.22 (0.92–1.60) | 0.161 | |
| | Q3 | 64 (31.2%) | 1.05 (0.79–1.40) | 0.734 | |
| | Q4 | 74 (36.1%) | 1.22 (0.93–1.61) | 0.153 | |
| Hard/lumpy stools | Q1 | 95 (45.9%) | 1.00 (reference) | — | 0.013 |
| | Q2 | 91 (44.4%) | 0.99 (0.80–1.23) | 0.947 | |
| | Q3 | 113 (55.1%) | 1.23 (1.01–1.49) | 0.037 | |
| | Q4 | 110 (53.7%) | 1.21 (1.00–1.48) | 0.056 | |
| Sensation of incomplete evacuation | Q1 | 39 (18.8%) | 1.00 (reference) | — | 0.005 |
| | Q2 | 49 (23.9%) | 1.30 (0.89–1.89) | 0.174 | |
| | Q3 | 53 (25.9%) | 1.40 (0.97–2.02) | 0.074 | |
| | Q4 | 61 (29.8%) | 1.64 (1.15–2.34) | 0.006 | |
| Sensation of anorectal blockage | Q1 | 33 (15.9%) | 1.00 (reference) | — | 0.027 |
| | Q2 | 49 (23.9%) | 1.55 (1.04–2.30) | 0.030 | |
| | Q3 | 44 (21.5%) | 1.40 (0.93–2.10) | 0.104 | |
| | Q4 | 51 (24.9%) | 1.64 (1.11–2.43) | 0.013 | |
| Manual maneuvers to facilitate defecation | Q1 | 7 (3.4%) | 1.00 (reference) | — | 0.555 |
| | Q2 | 4 (2.0%) | 0.61 (0.18–2.06) | 0.425 | |
| | Q3 | 9 (4.4%) | 1.33 (0.50–3.55) | 0.573 | |
| | Q4 | 7 (3.4%) | 1.11 (0.39–3.15) | 0.848 | |

All models: 0 vs 1–4; reference Q1; modified Poisson regression with HC3 robust covariance, adjusted for age group, sex, and home-cooking frequency.
